# Perforated Duodenal Diverticulum Treated Conservatively: Another Two Successful Cases

**DOI:** 10.1155/2017/4045970

**Published:** 2017-05-07

**Authors:** Jad A. Degheili, Mohammed H. Abdallah, Ali A. Haydar, Ahmad Moukalled, Ali H. Hallal

**Affiliations:** ^1^Division of General Surgery, Department of Surgery, American University of Beirut Medical Center, Riad El-Solh, Beirut 1107 2020, Lebanon; ^2^Division of Interventional Radiology, Department of Diagnostic Radiology, American University of Beirut Medical Center, Riad El-Solh, Beirut 1107 2020, Lebanon

## Abstract

Diverticula of the duodenum proceed those of the colon in respect to frequency of location. Incidence at times of autopsy ranges from 15 to 23%. Despite the fact that more than 90% of duodenal diverticulum cases are asymptomatic, complications if they do occur can be calamitous. Perforation is one of these rare complications. Surgical intervention has always been the mainstay for symptomatic/complicated duodenal diverticula, but with the advancement of imaging, medical treatment, and proper intensive observation, conservative treatment came forth. We hereby present two cases of duodenal diverticula, complicated by perforation and fistulization into the retroperitoneal cavity, both treated conservatively by Taylor's approach of upper gastrointestinal tract perforation. Review of other cases of duodenal diverticulum perforation has also been presented.

## 1. Introduction

Diverticulum of the duodenum is the second most common location after that of the large bowel. It has been on the bottom differential for chronic epigastric pain, abdominal bloating, nausea, and hyporexia, yet, almost 90% are asymptomatic. Complications if they do occur can lead to pancreatitis, hemorrhage, diverticulitis with or without perforation, and other biliopancreatic manifestations including cholidocholithiasis and cholangitis. Surgery has always been the mainstay approach for symptomatic diverticula. With the advent of new medical treatments and the high rate of morbidity postop, physicians are now trending more toward more conservative approach. What follows are another two successful cases of perforated duodenal diverticula treated conservatively.

## 2. Case One

An 81-year-old male was admitted to the Emergency Department with a sudden onset complaint of severe epigastric pain for the past four hours, high grade fever of 39,3°C, nausea, and vomiting. Albeit hemodynamically stable, he was tachycardic with a 105 bpm heart rate. Isolated epigastric tenderness without any signs of peritoneal irritation was elicited upon palpation. Laboratory workup revealed leucocytosis of 28,100 white blood cells with 95% left shift and lactic acid of 3.80 mmol/L. Remaining blood studies were normal, including liver function tests and amylase.

**Figure 1:**
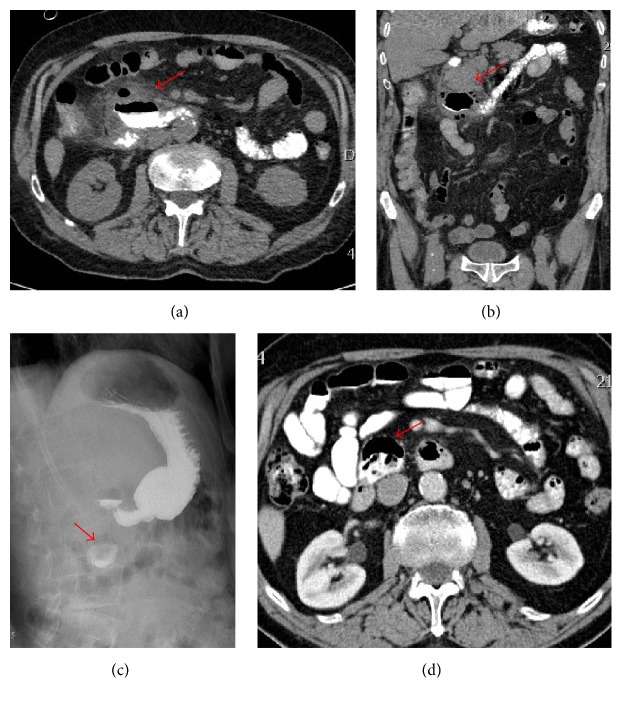
(a & b) Computed Tomography of the abdomen, with PO contrast, showing the evidence of a duodenal diverticulum with contrast extravasation, surrounded by fat stranding and air pockets (arrow), suggestive of perforation ((a) axial; (b) coronal). (c) Gastrografin swallow fluoroscopy, upon follow-up, showing the presence of contrast within the diverticulum (arrow) and absence of any extravasation. (d) Enhanced CT scan with IV and PO contrast showing the diverticulum with absence of extravasation and minimal air pocket with fat stranding (arrow).

A Computed Tomography (CT) scan of the abdomen/pelvis with intravenous and oral contrast showed the presence of a duodenal diverticulum measuring 4.5 × 3.0 cm, surrounded by fat streaking and multiple pockets of free air (Figures 1(a) and 1(b)), a constellation of findings consistent with a perforated duodenal diverticulum.

Patient was started on total parenteral nutrition (TPN) and broad-spectrum antibiotics. His clinical status improved gradually. Gastrografin swallow imaging followed by a CT scan, one week later, revealed contrast accumulation within a duodenal pouch, corresponding to the previously described diverticulum, with absence of contrast extravasation (Figures 1(c) and 1(d)). Clear fluid diet was started and advanced gradually as tolerated. He was discharged two weeks later with stable conditions.

## 3. Case Two

53-year-old female, with no antecedent medical or surgical history, recalled chronic episodes of epigastric pain, for which an esophagogastroduodenoscopy (EGD) and colonoscopy were done, 10 days prior to presentation, revealing a large duodenal diverticulum and multiple sigmoid diverticula. Two days after endoscopy, she underwent urgent surgical drainage of a large retroperitoneal collection with insertion of a Penrose drain within the right lower quadrant (RLQ). Upon transfer to our medical center, she was clinically stable, yet reporting occasional low-grade fever and alteration in consistency of the RLQ discharge to bilious in nature, over the past few days, with significant increase in its amount to around 1200 mL per 24 hrs. Physical examination was insignificant for any signs of peritonitis, but rather significant for biliary discharge from the RLQ drain.

Laboratory workup showed leukocytosis of 17,200 with 86% left shift; serum liver and pancreatic function tests were normal. Amylase and lipase level, from the draining fluid, were significantly elevated, measuring 482 IU/Lit and 11243 U/Lit, respectively.

**Figure 2:**
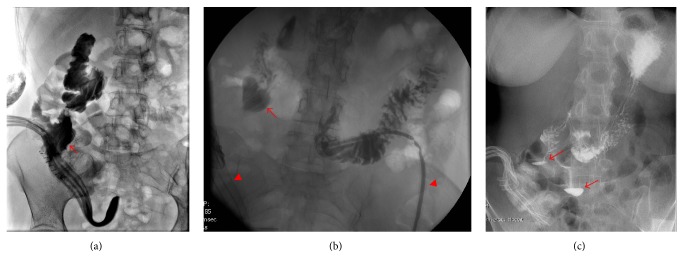
(a) Drainogram showing a fistulous tract (arrow) between the retroperitoneal cavity and the duodenum, secondary to a perforated duodenal diverticulum. (b) Gastrografin swallow fluoroscopy, upon follow-up, with absence of any contrast extravasation from within the duodenal diverticulum (arrow). Note the jejunostomy feeding tube and the retroperitoneal drain in place (arrow heads). (c) Layering of contrast, during a gastrografin swallow, into two duodenal diverticula (arrows), without any contrast extravasation, seen upon follow-up.

Fluoroscopic guided drainogram showed delineation of a retroperitoneal collection in the RLQ, with a fistulous tract in junction with a duodenal segment (Figure 2(a)), suggestive of a high output duodenal-retroperitoneal fistula. The fistula is likely secondary to diverticular perforation, after endoscopy. Patient was started on broad-spectrum antibiotics and TPN.

Gradually, her clinical status improved, and the output drainage started to decrease to around 350 mL/day. A feeding jejunostomy tube was then inserted and enteral feeding initiated (Figure 2(b)). Follow-up CT and gastrografin swallow imaging showed the evidence of two outpouching structures within the D2 and D3 segments of the duodenum with layering of contrast (Figure 2(c)), representing two wide-neck duodenal diverticula. Neither contrast collection within the peritoneal cavity nor any persistent fistulous tract was noted. She was then started on PO diet, which was advanced as tolerated. Forty days later, she was discharged home, off any drains.

## 4. Discussion

Duodenal diverticulum (DD) has first been described by the French pathologist Pierre Chomel in 1710, containing 22 gallstones [1]. Because of its rarity and because most are asymptomatic, it has been identified in only 5–10% of patients undergoing radiological or endoscopic procedures for other etiologies and in 15–23% at times of autopsy [2]. Around 60% of these diverticula are located in the second portion of the duodenum, within 2 to 3 cm from ampulla of Vater, referred to as perivaterian or periampullary diverticula. This is followed by 30% of diverticula located in the D3 portion, and around 8% present in the D4 segment [3]. Nearly 90% of diverticula are present on the medial surface (along the pancreatic or mesenteric border) of the duodenum, with multiple diverticula present in 10–15% of patients with this entity [4].

Two types of duodenal diverticula have been identified: the most common include the acquired extraluminal pseudodiverticulum, consisting of around 90% of duodenal diverticula, and the congenital intraluminal type which is rarer than the former type and occurs during early development [5]. 40% of cases having intraluminal diverticula are associated with other congenital malformations [6].

A surgical consensus states that intervention is only required for symptomatic duodenal diverticula, constituting only 1–5% of total cases [7]. This is attributed to the high complication rate that might occur after excision of the diverticulum [8]. For history, the first who performed diverticulectomy were Forssell and Key in 1915 [9].

Complications necessitating intervention for such diverticula can be categorized as follows: the most common are those related to biliopancreatic manifestations mainly cholidocholithiasis mostly pigmented stones [10], which may result in obstructing jaundice and cholangitis; mechanical obstruction of the common bile duct (CBD) by the diverticulum itself (Lemmel's syndrome) [8], or even acute pancreatitis if the pancreatic duct has been obstructed. Stasis of the bile within the CBD can thus result in malabsorption of vitamin B12 and steatorrhea. A second important complication includes inflammation or diverticulitis that may or may not end up with perforation: a fearful complication. Hemorrhage is also a serious complication of DD, especially if erosion occurs at the site of the pancreaticoduodenal arcade [11].

Searching the world English literature will reveal a near 171 cases [12–15] of perforated duodenal diverticula, since the first case described by Bassett in 1907. Most of these perforations (78%) are seen within the second portion of the duodenum, mainly along the medial wall, within 2 cm from the ampulla of Vater. Diverticulitis is the most common cause of perforation representing 62%, followed by enterolithiasis (~10%) [12]. Ulceration, iatrogenic causes [16], trauma [17], or even foreign bodies [14] are all rarer causes of perforation.

It is with great difficulty to differentiate between a perforated duodenal diverticulum and perforated duodenal ulcer; the former mostly involves distal portion of duodenum, whereas the later mostly involves the duodenal bulb [18]. Other differentials include peptic ulcer disease, colitis, retrocecal appendicitis, pancreatitis, or even cholecystitis [19].

In regard to treatment of duodenal diverticula, we should note that the clinical condition and hemodynamic stability of the patient guide the treatment: whether conservative versus surgical, and with surgical, this includes several options. A great importance and attention in case one decided for a surgical option is the location of the diverticulum in relation to the biliary system especially the ampulla of Vater. This can be aided by inserting a catheter through the ampulla by doing either a cholecystostomy or cholidostomy, intraoperatively [20, 21].

Given the high morbidity and mortality for the surgical options reaching as high as 30% [8], including duodenal leak and fistulization, the option of conservative treatment has become more and more implicated. The first nonoperative management of perforated DD had been reported by Shackleton in 1963 [22]. From 1963 to 1989, five new cases have been treated conservatively, two of which had duodenocolic fistulas. From 1989 to 2011, 14 (23%) out of 61 patients with perforated DD were successfully treated without operative interventions [12], the so-called “Taylor's approach for upper GI perforation,” mainly applied for duodenal ulcer perforation, which includes bowel rest with or without nasogastric tube suction, intravenous hydration and antibiotics, total parenteral nutrition, and, when needed, percutaneous catheter drainage of retroperitoneal collections [23].

In the Thorson et al. series [12] of 61 patients presenting with perforated DD, 47 (77%) underwent operative treatment versus 14 (23%) who underwent successful nonoperative management. The complication in the surgical group was reported in 17 (36%) out of 47 patients, versus only 1 (7%) complication seen in patients undergoing nonoperative management. Mortality in surgical group was 3 (6%) out of 47 versus null in the conservative group.

## 5. Conclusion

We hereby reported another two successful cases of conservative management of a perforated duodenal diverticulum. Surgical approach has long been the preferred option for most surgeons, yet with advancements in all medical specialties, physicians are leaning more toward less invasive approaches, given the more info we had from previous case series, aiming for avoiding any drastic postop complications.

## Abbreviations

DD: Duodenal diverticulum(a)
CT: Computed Tomography
TPN: Total parenteral nutrition
EGD: Esophagogastroduodenoscopy
CBD: Common bile duct.
